# A Survey on Banknote Recognition Methods by Various Sensors

**DOI:** 10.3390/s17020313

**Published:** 2017-02-08

**Authors:** Ji Woo Lee, Hyung Gil Hong, Ki Wan Kim, Kang Ryoung Park

**Affiliations:** Division of Electronics and Electrical Engineering, Dongguk University, 30 Pildong-ro 1-gil, Jung-gu, Seoul 100-715, Korea; ljwgs@dongguk.edu (J.W.L.); hell@dongguk.edu (H.G.H.); yawara18@hotmail.com (K.W.K.)

**Keywords:** banknote recognition, counterfeit banknote detection, serial number recognition, fitness classification, various sensors

## Abstract

Despite a decrease in the use of currency due to the recent growth in the use of electronic financial transactions, real money transactions remain very important in the global market. While performing transactions with real money, touching and counting notes by hand, is still a common practice in daily life, various types of automated machines, such as ATMs and banknote counters, are essential for large-scale and safe transactions. This paper presents studies that have been conducted in four major areas of research (banknote recognition, counterfeit banknote detection, serial number recognition, and fitness classification) in the accurate banknote recognition field by various sensors in such automated machines, and describes the advantages and drawbacks of the methods presented in those studies. While to a limited extent some surveys have been presented in previous studies in the areas of banknote recognition or counterfeit banknote recognition, this paper is the first of its kind to review all four areas. Techniques used in each of the four areas recognize banknote information (denomination, serial number, authenticity, and physical condition) based on image or sensor data, and are actually applied to banknote processing machines across the world. This study also describes the technological challenges faced by such banknote recognition techniques and presents future directions of research to overcome them.

## 1. Introduction

### 1.1. Motivation of the Research

Despite a decrease in the use of currency due to the recent global expansion in electronic financial transactions, transactions in real money continue to be very important in the global market [1]. While performing transactions in real money, touching and counting notes by hand is still a common practice in daily life, but the use of various types of automated machines has become essential for large-scale and safe transactions. Such automated self-service machines include automated teller machines (ATMs) for money deposits and withdrawals, as well as financial transactions [2], banknote counters [3] and coin counters [4], mostly used in banks, and automatic vending machines, into which money is inserted to purchase goods [5]. These devices must be equipped with four essential functions: banknote recognition, counterfeit banknote detection, serial number recognition, and fitness classification. While limited surveys have been conducted in previous studies on the areas of banknote recognition and counterfeit banknote recognition, this paper is the first survey of its kind to review all four areas. This lack of research is ascribable to the fact that banknote recognition studies have been mostly carried out in industrial settings rather than for academic purposes. In this study, previous studies and the advantages and disadvantages of the different methods related to the four aforementioned areas used therein are analyzed, and future applications are also presented.

### 1.2. Scope and Method of Our Research

#### 1.2.1. Scope of Our Research

This paper presents methodologies for recognizing banknotes in the four main categories of banknote recognition, counterfeit banknote detection, serial number recognition, and fitness classification.

Banknote recognition generally concerns classification of banknotes by denomination, i.e., the currency amount of a note of a specific country. This classification also enables recognition of the year of printing and input direction of the classified denomination. In some studies, the scope of recognition is extended to simultaneous recognition of two or more national currencies. Technologies for banknote recognition are described in detail in Section 2.

**Figure 1:**
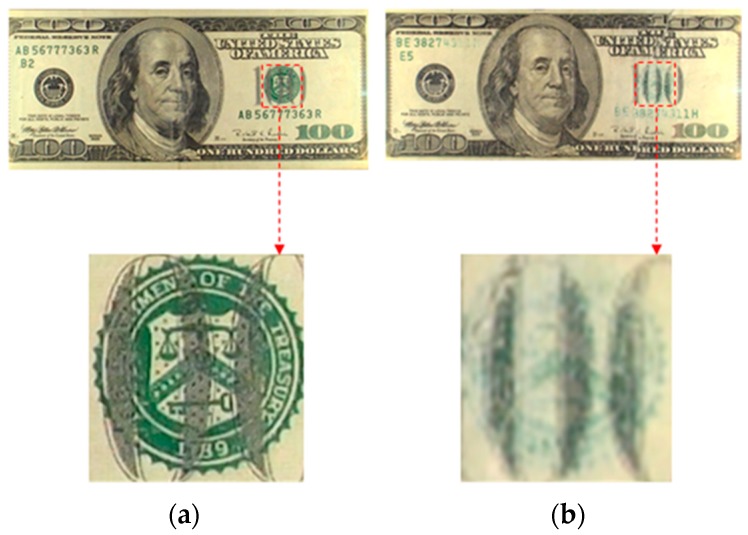
Example of genuine and counterfeit banknotes (USD 100 bill): (**a**) a genuine banknote; (**b**) a counterfeit banknote.

Counterfeit banknote detection generally concerns methods for distinguishing between genuine and fake notes. As shown in the example of a genuine and a counterfeit USD 100 bill in Figure 1a,b, respectively, a validation check is done by examining anti-counterfeiting features. Section 3 describes technologies for counterfeit banknote detection in detail.

**Figure 2:**
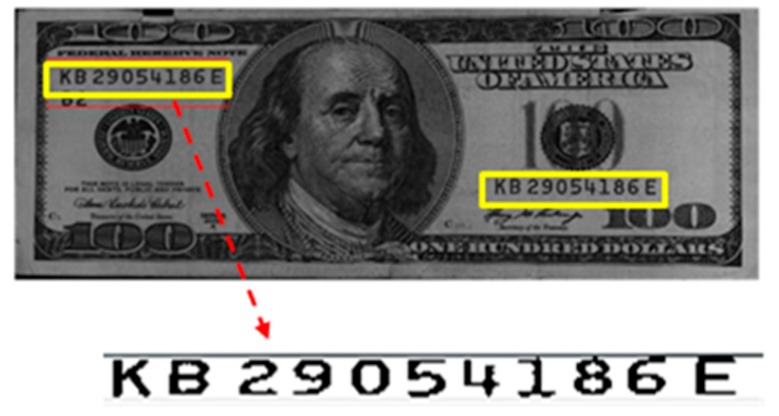
Example of serial number code (USD 100 bill).

A banknote serial number is a unique alphanumerical identifier engraved on each banknote in the banknote production process. It contains the name of the issuing bank and serial information of each denomination [6]; Figure 2 shows the serial number of a USD 100 bill. Since each banknote has its own unique serial number, it can be used to trace its source and circulation route and can thus be efficiently used to detect counterfeit banknotes. Related technologies are described in detail in Section 4.

**Figure 3:**
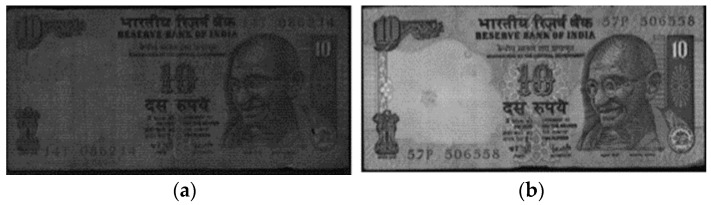
Example of unfit and fit banknotes (INR 10 bill): (**a**) Unfit banknote; (**b**) Fit banknote.

Fitness classification of banknotes generally concerns methods for classifying banknotes according to their physical conditions, such as soiling. As shown in the example of two INR 10 bills in Figure 3, banknotes of the same denomination may exhibit fit or unfit conditions, which include soiling and creases (Figure 3a), depending on circulation intensity and climate conditions. In order to maintain the fitness of banknotes in circulation, automated self-service terminals, such as ATMs, need to be equipped with a fitness classification function to sort out and retrieve unfit banknotes. Retrieving unfit banknotes is also necessary for preventing banknote classification errors. Fitness classification-related technologies are described in detail in Section 5.

#### 1.2.2. Method of Our Research 

**Figure 4:**
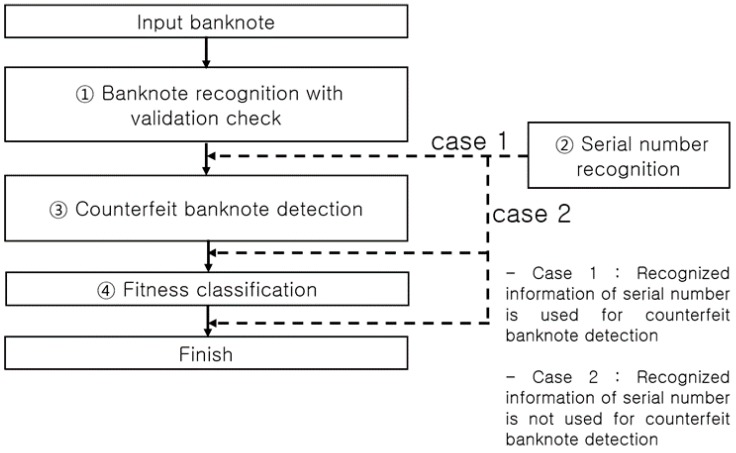
Banknote recognition process flow in an automated device.

Figure 4 presents a typical process flow of banknote recognition implemented in a self-service terminal. An input banknote is scanned by a sensor to discern the image and other data necessary for recognizing its denomination and anti-counterfeiting features. The first step in the process of banknote recognition is identifying the denomination of an input banknote, thereby clustering all classes into a single class, and implementing a size-based ① validation check, followed by ① banknote recognition on the class of notes identified as banknotes, ③ counterfeit banknote detection using anti-counterfeiting data specific to each recognized denomination, and ④ fitness classification. The flow direction of ② serial number recognition is determined depending on the counterfeit banknote detection method as shown in Figure 4. System efficiency is the main reason for setting the process flow direction as ① validation check and banknote recognition, then ③ counterfeit banknote detection, and finally ④ fitness classification. For example, any input materials other than banknotes to be classified, such as common paper and newspaper, are sorted out at the ① validation check step, obviating the later steps of ③ counterfeit banknote detection, ② serial number recognition, and ④ fitness classification. On the other hand, an input counterfeit banknote similar to the classified banknote [7] may pass through the banknote recognition step, but will likely be detected as counterfeit in the counterfeit banknote detection step implemented using anti-counterfeiting features specific to the denomination classified, which makes the steps of serial number recognition (case 2 in Figure 4) and fitness classification superfluous.

In a common embedded system environment, such as a banknote counter, rapid real-time recognition processing is required so that it can be implemented concurrently with banknote counting. When performing counterfeit banknote detection, for example, unnecessary computation should be excluded to rapidly process the banknotes queued for input. There are also cases in which steps such as serial number recognition and fitness classification need to be processed in the on/off mode to enhance the computational flow efficiency. Taking into account such processing paths, most automated currency recognition machines use the processing algorithm presented in Figure 4. Therein, however, the ② serial number recognition processing step can be bifurcated (case 1 or 2) depending on whether the serial number information is used for counterfeit banknote detection or not. If the serial number information is used for counterfeit banknote detection (case 1), the input banknote goes through the steps of serial number recognition for the corresponding denomination and counterfeit banknote detection. On the contrary, if the serial number information is not used for the counterfeit banknote detection (case 2), the time-consuming serial number recognition operation can be performed after the counterfeit banknote detection step, thus reducing the time taken for counterfeit banknote detection. As regards the serial number recognition and fitness classification functions, given their less important roles compared to the banknote recognition and counterfeit banknote detection functions, they could be removed from the main computational flow and processed in the on/off mode. In the off mode of the serial number recognition and fitness classification functions, the user can perform the computations for counterfeit banknote detection more rapidly. This method of enabling the off mode of the serial number recognition and fitness classification functions can be essential for a banknote counter that carries out real-time computations of over 1000 notes per minute to perform rapid counterfeit detection.

Section 2 describes the aforementioned image-based banknote recognition methods. Section 3 describes counterfeit banknote detection of the recognized denomination on the basis of the obtained image and sensor data (ultra violet (UV), near-infrared (NIR), etc.). Section 4 describes methods for image-based serial number recognition of a denomination, and Section 5 describes image-based fitness classification of the soiled condition of a denomination recognized as a genuine banknote. Lastly, Section 6 presents methodologies employing the described banknote recognition technologies and areas for further study and future directions.

## 2. Banknote Recognition

### 2.1. Banknote Recognition Methodology

**Figure 5:**
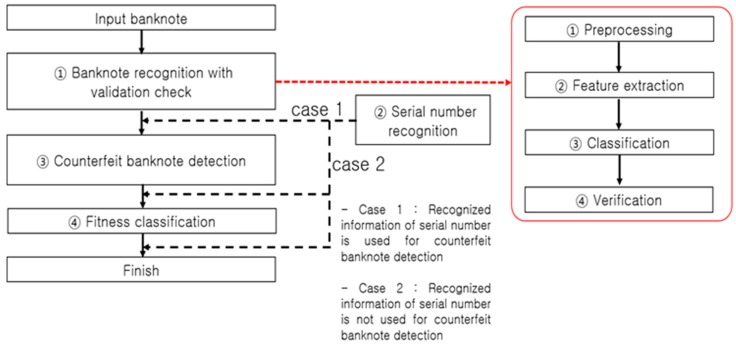
Banknote recognition process flow.

Banknote recognition is a process step in which the denomination (e.g., $1, $10 and $100), direction (e.g., forward or backward direction), and side (e.g., obverse or reverse side) of the input banknote are classified. The reason for classifying direction and side in addition to denomination is that the position of a region of interest (ROI) within a banknote, which is used to implement the later process steps (serial number recognition, counterfeit banknote detection, and fitness classification), changes according to the direction and side of the banknote, as shown in Figure 5. For example, if the ROI of a $1 bill is in the top-left corner of its image when input in the forward direction, the position changes to the bottom-right corner when input in the backward direction. Moreover, the function of an automated machine of classifying input banknotes by denomination, direction, and side is essential for a bank employee to manually check the automatically classified banknotes.

Most conventional sensors for banknote recognition are visible-light line sensors, such as contact image sensors (CIS) [8,9,10,11,12,13]. Such sensors mostly obtain color or black-and-white images and are used for the typical process flow of ① preprocessing, ② feature extraction, ③ classification, and ④ verification, as shown in Figure 5. Using a color image sensor is more advantageous because it provides more information than a black-and-white sensor [14], but its production cost is higher. Besides image resolution, national currencies and classification criteria are factors influencing the efficiency of banknote recognition.

Table 1 lists previous studies on banknote recognition of various national currencies. In this table, “N/I” means the case that the appropriate information is not provided in the paper. “A” represents the case that the database is available for research purpose whereas “N/A” shows the case that the database is not available. USD, CNY, INR, and EUR are the most frequently studied currencies. In Table 1, the largest numbers of images are included in the database of [11]. Most databases are not available, except for that described in [14].

A number of studies have also been conducted on simultaneous recognition of multiple national currencies. The number of classes to be classified increases with the number of national currencies to be simultaneously recognized, and the classification efficiency decreases with the increase in the number of the national currencies to which the same algorithm is applied. If a banknote counter can recognize notes with a speed exceeding 1000 notes per minute, a complicated system such as a multi-currency simultaneous recognition processing system requires a recognition algorithm optimized for accuracy and speed. It involves the process steps presented in Figure 5: ① preprocessing: image preprocessing such as precise banknote region segmentation, optimal dimension reduction, and noise removal; ② feature extraction: extraction of features best-suited for the classification of the given denomination; ③ classification: classification of the recognized denomination into classes using the extracted features and classifier; and ④ verification: recheck of the classified denomination).

### 2.2. Preprocessing of Banknote Image

**Figure 6:**
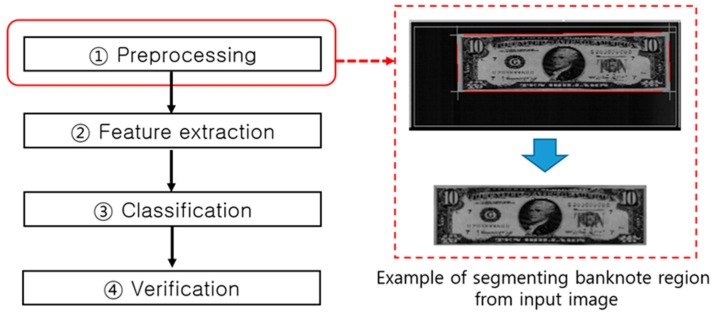
Preprocessing step in the banknote recognition process flow.

Preprocessing of the images obtained through the sensor involves the following process steps: banknote region segmentation, as shown in Figure 6, to extract the precise denomination region [8], noise removal and gray level reduction, brightness normalization and contrast enhancement, and reduction of the image resolution and number of image channels to reduce the computational burden.

First of all, the banknote region segmentation algorithm is applied to extract the precise banknote region. Relevant methods include corner detection [8,9], least square methods and fuzzy systems [12], and component labeling using the Y component of the YIQ color space via axis transformation [68] as shown in Table 2. If a banknote has been in circulation for a long time, it may be difficult to extract accurate banknote recognition features due to its surface being soiled by dirt and sebum from users’ hands. To address this, noise removal is performed as a general preprocessing step using techniques based on the Wiener filter [10,49,55,65,71] or median filter [42,64]. Noise occurring in the imaging process or banknote aging can also be diminished by reducing the gray level of the image beyond the 0–255 range [54,65,71,72]. Some studies have presented methods to normalize the brightness and improve the contrast of the image by means of histogram equalization [42,45].

Finally, a data reduction process is performed in order to reduce the computational burden. As mentioned previously, this process is essential because of the limited memory and processing speed, although the larger the amount of information, the greater the performance. In general, image interpolation, e.g., the nearest neighbor interpolation method [10,67], is used to reduce image size. Furthermore, conversion from RGB to gray scale can be performed to reduce the color dimension [23,39,46,55,63,72].

### 2.3. Feature Extraction

**Figure 7:**
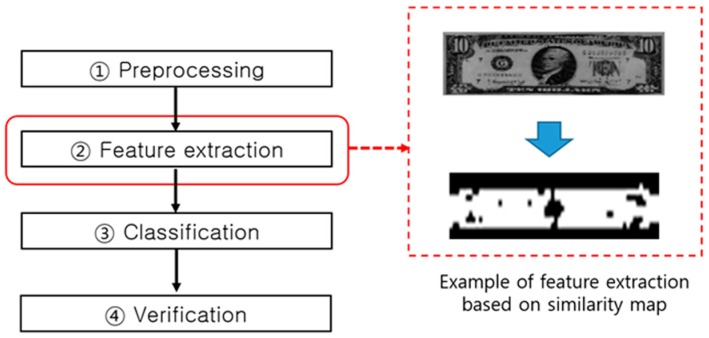
Feature extraction in the banknote recognition process flow.

As shown in Figure 7, the preprocessed data undergoes feature extraction designed to facilitate the denomination classification. Figure 7 provides an example of an extracted similarity map expressing the feature regions for efficient classification.

Among the methods for extracting banknote features to classify denominations presented in Table 3, those using the size or length data of a banknote as a parameter [45,56,60,65,68,72] are especially useful for classifying national currencies with different sizes or lengths for different denominations. There are also methods using RGB, HSV, or features in the HSI color space [37,40,45,49,50,61,68], methods using edge-based features expressed with Canny, Prewitt, or Sobel operators [40,44,54,60], and methods using histogram information-based features such as correlation, central moments, kurtosis, mean, standard deviation, and skewness [39,43,53,59,64,65].

Furthermore, there are methods that use texture features extracted by local binary patterns (LBP) [41,49] and features based on the values of the gray-level co-occurrence matrix (GLCM) [39,53,64]. Conventional methods of feature extraction widely used in the common pattern recognition fields, include methods using principle component analysis (PCA) [8,9,15,20,21,22,23,26,46] and linear discriminant analysis (LDA) [43,46,70], and also methods using genetic algorithm (GA)-based learning to identify the feature mask optimized for the target class [24,30,69,73]. Similarity maps or difference maps are an automated optimal feature search method [9,10,19].

Among high-performance methods for banknote feature extraction, there are wavelet transform-based methods, with a high computational burden being their drawback [11,16,44,47,48], and methods using the scale-invariant feature transform (SIFT) or speeded up robust features (SURF) algorithm, known to be robust to scale and rotation changes [14,17,18,25,35,36,61,67,74], which have hence been used as methods of feature extraction for banknote recognition. The method based on compressed sensing is known to be useful for data dimension reduction [27]. Features can be extracted using information based on optical character recognition (OCR) as well [38]. Lastly, many methods use selected ROIs, instead of the entire banknote region, as feature extraction regions [8,9,13,17,20,23,30,32,36,38,39,43,48,59,68].

### 2.4. Classification and Verification

**Figure 8:**
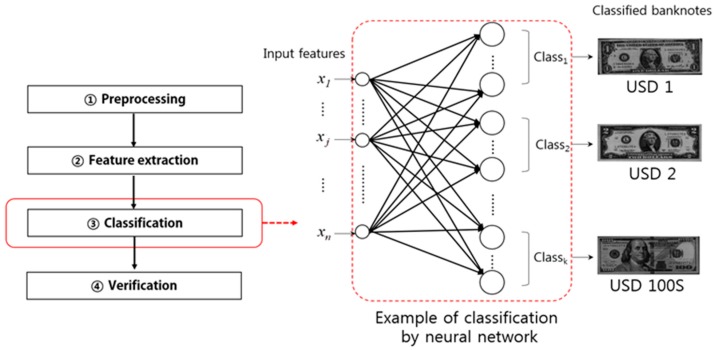
Classification example in the banknote recognition process flow.

The neural network (NN)-based method [15] shown in Figure 8 is the representative method for input banknote classification using the features extracted in the feature extraction step described in Section 2.3.

As shown in Table 4, NN-based methods for classifying input banknotes use various neural networks based on learning vector quantization (LVQ), ensemble networks (ENN) using negative correlation, and probabilistic neural networks (PNN) [15,16,20,21,22,24,26,29,30,31,32,33,34,38,45,46,47,49,53,54,56,57,58,62,63,66,69,72,73].

Among other classification methods listed in Table 4, there are simple methods of comparing distances using the Euclidean distance-based classifier [36,37,41,42,48,51] or the Mahalanobis distance-based classifier [23], and complicated methods using sophisticated pattern recognition-based classifiers, such as support vector machines (SVM) [8,11,39,43,67,71] or hidden Markov models (HMM) [13,65,71]. Furthermore, there are methods using the clustering-based K-means classifier [8,9] and denomination classification methods using K-NN (*k*-nearest neighbors) [55,64].

There are also methods for classifying denominations in which preclassification, based on the banknote input side, direction, size, or a Gaussian mixture model (GMM), is performed before proceeding with denomination recognition on the preclassified banknote with a reduced number of classes for matching [8,10,32,75]. This approach can greatly enhance classification accuracy and rapidity because it reduces the number of classes at the preclassification step rather than performing the denomination and side/direction check on all input banknotes.

**Figure 9:**
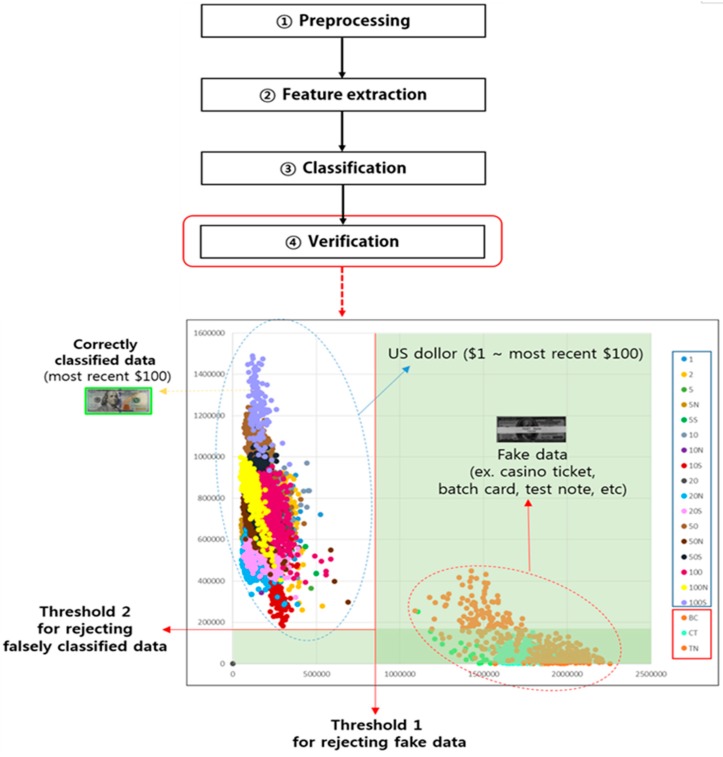
Verification example in the banknote recognition process flow.

There are also methods in which the denomination classification errors are reduced by performing verification of the classified banknotes [9,54]. Figure 9 illustrates an example of the verification process in which fake banknote recognition errors and denomination classification errors in the results of classification performed using the Euclidean distance or K-means classifier are removed using threshold 1 based on the distance from the first matched candidate class (located at the shortest distance) and threshold 2 based on the matching distance between the first candidate class (located at the shortest distance) and the second candidate class (located at the second-shortest distance).

Counterfeit banknote recognition errors can be removed as follows: a counterfeit note, such as a casino ticket, batch card, or test note, may be judged a genuine banknote as the first candidate class in the denomination classification step, but because the distance from the first candidate class is shown to be larger compared to that of a genuine banknote, exhibiting a distance going beyond threshold 1 when matched, it is thus rejected as a fake banknote. This is illustrated in Figure 9.

Denomination recognition errors can be reduced as follows: if the difference in the distance between a banknote matched as the first candidate class in denomination classification and the second candidate class is smaller than threshold 2, as shown in Figure 9, the result of the denomination classification for the matched banknote is judged to be unreliable and the matched result is rejected.

### 2.5. Analyses and Discussion of Banknote Recognition

Image-based banknote recognition generally uses color images obtained in the visible light spectrum [17,18,23,32,38,39,40,42,45,49,55,60,61,63,68] and undergoes general image recognition processes, such as preprocessing, feature extraction, classification, and verification. From the existing body of literature dealing with banknote recognition, important studies were selected and presented in Table 5. Selection criteria were the processing time of the digital signal processor (DSP) environment actually used by the banknote counter, ≥95% recognition rate based on a data size of ≥10,000 notes, and significant results yielded in various class environments, such as multi-currency simultaneous recognition.

In one study [8], in which an experiment was performed on over 60,000 USD denomination bills in a real banknote counter DSP environment, counting performance of 15.6 ms per note with a recognition rate of 99.886% was demonstrated. In the method used in this study, the input banknotes first undergo classification by input side and direction, followed by denomination classification. The salient feature of this method is progressive processing depending on the number of classes to be classified, thus optimizing the processing efficiency. In another study [73] conducted in a real banknote counter DSP environment, an error-free recognition rate of over 97% was achieved on 100,000 USD and JPY denomination bills by adopting the method of selecting a GA-based optimal mask and applying it to a NN. This method was also adopted in a study [69] in which a 97% recognition rate was achieved in simultaneous denomination recognition of four national currencies. The methods used in these studies may well be recommended for banknote counters. The method of another study [9] achieved superior results compared to the method used in one of the above-mentioned studies [8] in terms of processing speed and use of memory capacity, also using the method of processing in a real DSP equipment environment, yielding a classification accuracy of 99.998% on over 90,000 USD denominations. Its particular significance lies in the fact that it extracts an ROI of a banknote automatically without manual selection using a similarity map. Another study [10] presented a method for simultaneously recognizing five national currencies (USD, EUR, KRW, CNY, and RUB), demonstrating 100% accuracy in recognizing 84,800 experimental banknotes without error. Study [16] presented a method using frequency characteristics regarding the directionality of a banknote based on the quaternion WT and confirmed its performance (≥99% on average) on 15,000 notes each of USD, EUR, and CNY notes each. Study [65] presented a method of simultaneously recognizing 23 different national currencies, including USD, EUR, INR, and CNY, using the note’s size information and a HMM to model its texture characteristics, and demonstrated a classification accuracy of 98% on 150 notes per denomination.

Study [67] presented a method for an actual banknote ATM DSP environment. With a per-note processing time of 54 ms, it performed simultaneous recognition of USD and EUR and demonstrated a classification accuracy of higher than 99.8%. This method uses the dense SIFT algorithm for feature extraction and is significant as an approach to improving the robustness of the processing rate of the SIFT algorithm against scale change. Study [19] presents a real-time processing method in an embedded system capable of a processing speed of 16 ms per note using the ROI showing the largest inter-class difference on a difference map and GLVQ-based classification with an accuracy of 99% on 65,700 USD bills. In the method presented in Study [69], a GA-based optimal mask is selected and applied to a NN, as in Study [73] mentioned above. It demonstrated a multi-currency simultaneous classification accuracy of 97% on 20,000 JPY, ITL, ESP, and FRF notes using a real banknote counter. Study [74] presented a multi-currency simultaneous recognition method combining a mobile camera and server communication system. It performs complicated recognition operations using a high-performance server computer, thus reducing processing time and enhancing performance. It showed a multi-currency simultaneous recognition accuracy of 95% on several national currencies, including INR, CNY and EUR. The following issues should be dealt with and solved in the studies of banknote recognition:

- The banknote recognition function of a banknote counter should ensure not only a stable recognition rate, but also real-time processing speed because it continuously handles real money.
- The per-note processing time should be constant because time discrepancy in processing individual notes leads to non-normal storage of continuous high-speed banknote data input, triggering a system crash.
- With the increasing demand for simultaneous multi-currency recognition, stable recognition and a rapid processing speed for an increased number of classes are required, unlike the initially used manual selection-based single-currency recognition methods.
- While there is a considerable body of research presenting numerous banknote recognition methods using feature extraction and classifiers, no study has yet been conducted on the convolutional neural network (CNN)-based banknote recognition, which has recently been attracting attention. This may be ascribed to the difficulty associated with loading a high-performance graphics card capable of the parallel processing essential for high-speed CNN processing onto a banknote counter. Therefore, this method may be applied to server-based high-capacity counting systems in the future.

## 3. Counterfeit Banknote Detection

### 3.1. Counterfeit Banknote Detection Method

#### 3.1.1. Analyses of Anti-Counterfeiting Features inside a Banknote

**Figure 10:**
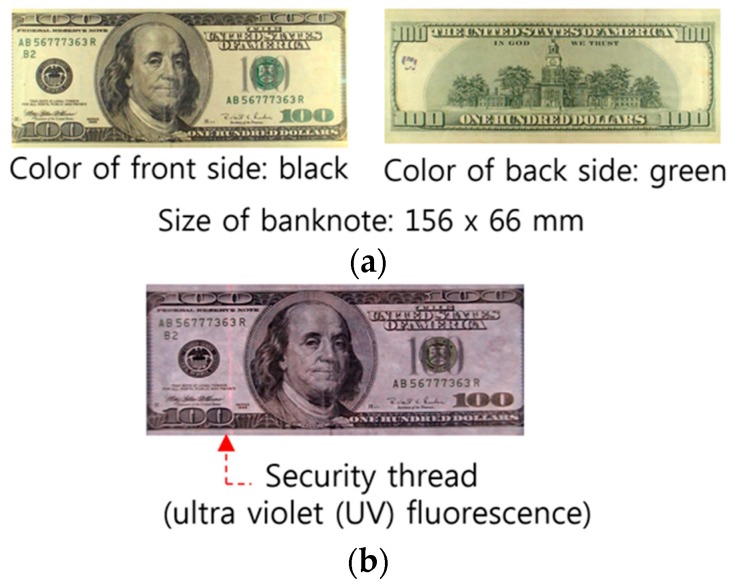
Visible light reflection image and ultraviolet fluorescence factor on a USD 100 bill: (**a**) The visible light reflection image of the recent USD $100; (**b**) Anti-counterfeiting feature.

As presented in Figure 5, banknote recognition is followed by counterfeit banknote detection. Most banknotes contain various anti-counterfeiting features. Figure 10 shows images of visible light reflection and ultraviolet fluorescence on a genuine USD 100 bill. Examples of anti-counterfeiting features are color, size, and security threads [76].

**Figure 11:**
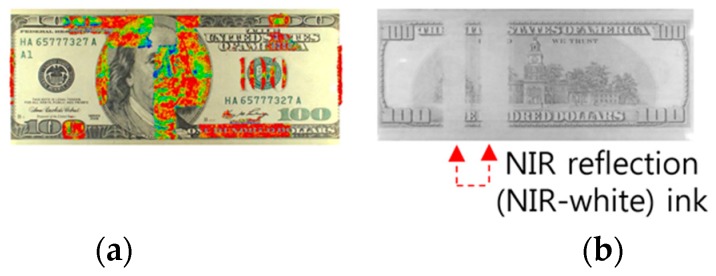

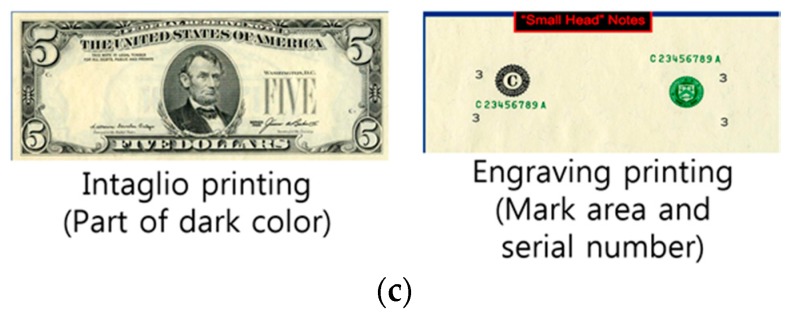
USD anti-counterfeiting features (magnetic factor, IR factor, and printing technology): (**a**) Magnetic factor for counterfeit prevention; (**b**) NIR factor for counterfeit prevention; (**c**) Printing scheme for a genuine banknote USD.

Figure 11 highlights the anti-counterfeiting features of a genuine USD 100 bill: the magnetic factor (obverse), two anti-counterfeiting lines responding to IR reflection, and intaglio and engraving technologies (reverse). As illustrated in Figure 11a,b, the magnetic factor and NIR factor contained in a genuine banknote, which are not visible under visible light, are perceived by a magnetic sensor and NIR sensor, respectively. Therefore, counterfeit banknotes without such magnetic and NIR factors can be easily detected by the aforementioned sensors. Figure 11c illustrates the printing technologies used in a genuine USD banknote: the dark-colored parts undergo intaglio printing and the mark or serial number undergo engraving printing. If a counterfeiter applies intaglio or engraving printing to the entire banknote, it can be detected as a counterfeit banknote due to a printing state different from a genuine banknote [76].

**Figure 12:**
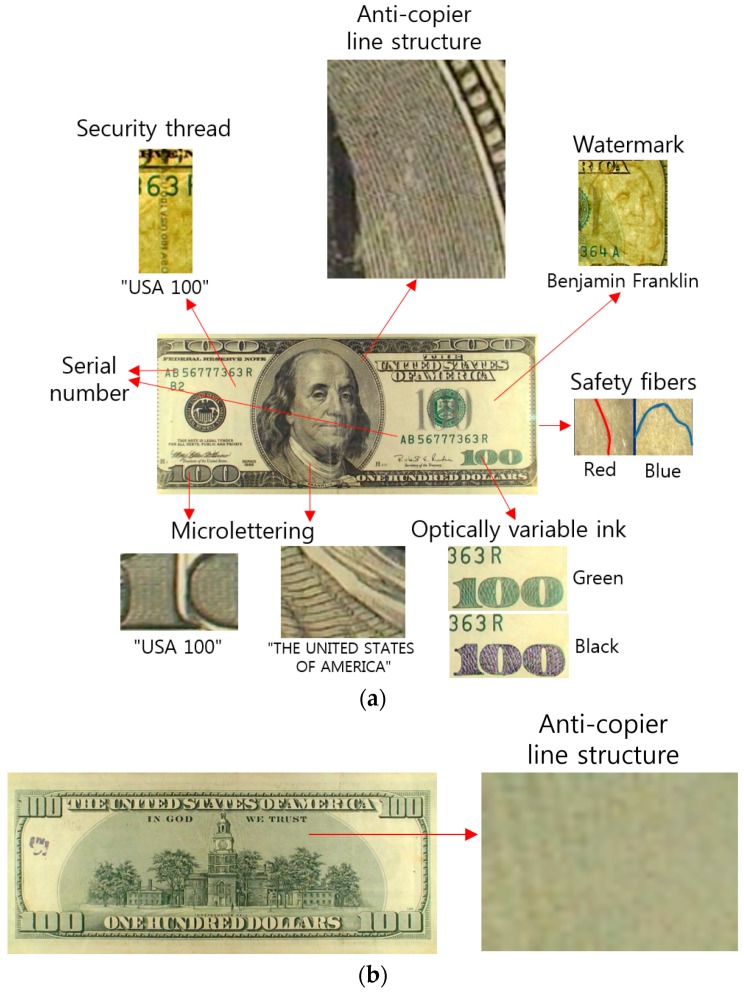
Analysis of anti-counterfeiting features inside a USD 100 bill: (**a**) Front side; (**b**) Back side.

Figure 12 shows other anti-counterfeiting features on the obverse and reverse sides of a genuine USD 100 bill: a security thread, anti-copier line structure (fine line printing), a watermark, safety fibers, optically variable ink, microlettering (microprinting), and serial numbers. The optically variable ink appears to change color depending on the viewing angle. Microlettering (microprinting) refers to microfine printed letters of specific words, such as “USA 100” and “THE UNITED STATES OF AMERICA” in the corresponding parts (Figure 12a). These anti-counterfeiting features are used in the detection of counterfeit banknotes [76,77,78,79].

Security features for detecting counterfeit banknotes can be called machine readable security features because they can measured by counterfeit detection machines [80]. In previous research [81], various techniques for printing the security features on genuine banknotes have been reported. These features are different for different denominations, and the security features of EUR are reported in [82].

#### 3.1.2. Counterfeit Banknote Detection

As presented in Table 6, there are a variety of methods for identifying anti-counterfeiting features by extracting features related to brightness information [83,84], fluorescence characteristics [85,86,87,88,89,90,91,92], fidelity of the serial number and printing [93,94], and security threads [95]. Simple methods using only wavelengths of the visible light spectrum have limitations in accurately identifying anti-counterfeiting features. Sensors with various spectral ranges to sense UV [85,86,96,97] and IR [75,82,97,98,99,100,101] wavelengths, X-ray, [88] etc. are required.

**Figure 13:**
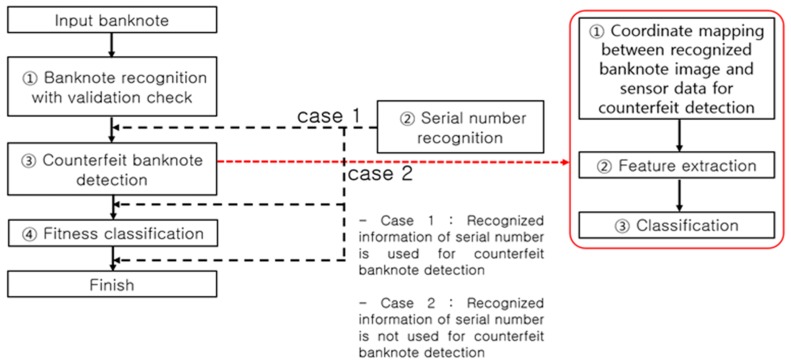
Counterfeit banknote detection process flow.

Most of the counterfeit banknote detection methods presented in previous studies corresponding to step ③ in Figure 13 (left), are carried out based on denomination and banknote input information (input side, deflection, inclination, etc.) after the banknote recognition step. As mentioned above, the process flow of counterfeit banknote detection bifurcates into the use and non-use of the serial number (Figure 13 case 1 and case 2, respectively).

In Figure 13, ③ counterfeit banknote detection in the overall process flow (left) follows the subprocess steps of ① coordinate mapping between the recognized banknote image and sensor data for counterfeit detection, ② feature extraction in which ROIs are selected from the recognized banknote image for anti-counterfeit feature extraction and sensor data are extracted for counterfeit detection based on the related coordinate information, and ③ classification through which counterfeit banknotes identified using the detection features are thus extracted.

As was the case with banknote recognition, anti-counterfeiting features for counterfeit banknote detection vary for individual denominations of the national currency concerned, and their efficiencies may also vary by denomination. Table 7 lists studies that have been conducted on counterfeit banknote detection by national currency. In this table, “N/I” means the case that the detail information is not shown in the paper. “N/A” represents the case that the database is not available for research purpose. The largest number of studies on counterfeit banknote detection focus on the Indian rupee, followed by the Euro and US dollar. In Table 7, the largest numbers of images are included in the database of [98]. All the databases are not available because the available counterfeit banknote can be illegally used.

### 3.2. Coordinate Mapping between Recognized Banknote Image and Sensor Data for Counterfeit Detection

**Figure 14:**
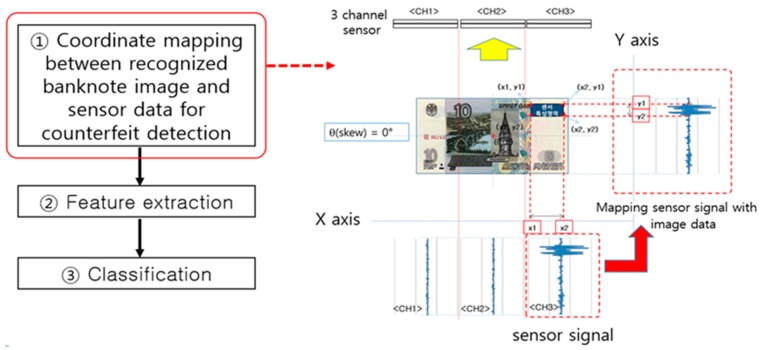
Example of data matching between banknote image and sensor data in the counterfeit banknote detection process flow.

Methods for counterfeit banknote detection are usually applied after the banknote recognition step for the validation check of the input banknote by judging the genuineness of each feature based on the input denomination and input information (input side, direction, deflection, inclination, etc.), combining such input data with IR, UV, and MG sensor data related to individual anti-counterfeiting features. Figure 14 illustrates an example of matching a banknote image and sensor signals around the ROIs for anti-counterfeiting features when a note recognized as a genuine banknote is input.

### 3.3. Feature Extraction

**Figure 15:**
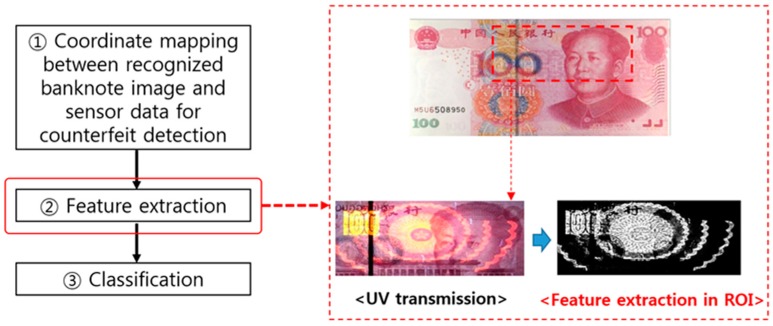
Example of UV anti-counterfeiting feature extraction within an ROI in the counterfeit banknote detection process flow.

Anti-counterfeiting features can be extracted using the sensor signals matched in predetermined ROIs, as shown in the example of extracting a feature using the UV-scanned signal of the corresponding ROI presented in Figure 15. Likewise, various methods listed in Table 6 are employed for counterfeit detection using light of various spectral ranges besides visible light or MG information.

Table 8 lists various methods for extracting anti-counterfeiting features, such as intaglio printing, ink properties, artwork, fluorescence, or year of printing-based feature extraction [87,106], bit-plane slicing and canny edge detection [116], watermark-based feature extraction [105,106,108,110,113,114], and luminance histograms and texture features from GLCM [84]. As shown in Table 7, the Indian rupee INR has been most intensively studied with respect to counterfeit detection, frequently by using methods for extracting features related to intaglio printing, ink properties, artwork, fluorescence, year of printing, watermarks, security threads, optically variable ink, identification marks, number panels, microlettering or latent images. With the continuing trend of increasingly sophisticated counterfeit banknotes, studies on more accurate counterfeit banknote detection methods based on multi-features are underway.

If a banknote is in circulation for a long time, it may be difficult to extract correct features for counterfeit banknote detection due to its surface being soiled by dirt and sebum from users’ hands. To solve this problem, noise removal is performed as a general preprocessing step using techniques based on median filtering [87,116], intensity thresholding [97], frame averaging [98], Gaussian low-pass filtering [120], and truncated-inhomogeneity-value with squared-homogeneity-difference [75]. Some studies have presented methods to normalize the brightness and improve the contrast of the images [116,117].

### 3.4. Classification of Counterfeit Banknote

**Figure 16:**
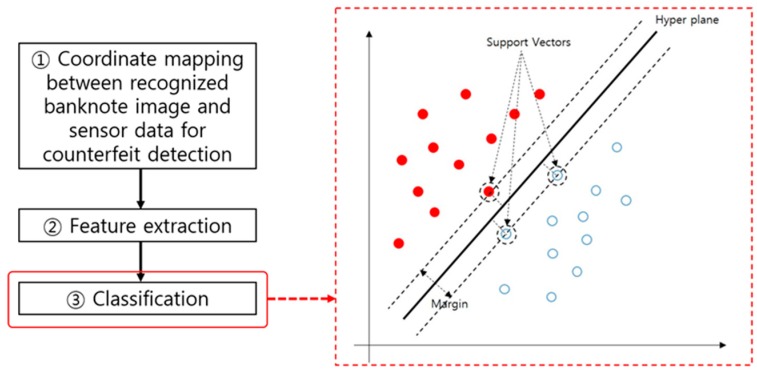
SVM-based counterfeit banknote classification in the counterfeit banknote detection process flow.

Classification, the last step in counterfeit banknote detection, is carried out using the anti-counterfeiting features extracted in the previous step. Methods used for counterfeit classification are similar to those used for classifying banknote recognition. Unlike banknote recognition, the two classes are genuine banknote and counterfeit banknote. Figure 16 illustrates an example of SVM-based counterfeit banknote classification.

As listed in Table 9, counterfeit banknote classification is performed using various algorithms such as template matching [106] or keypoint matching [117], an artificial NN [121], an SVM [84,87,119] or multiple kernel SVM [111].

### 3.5. Analyses and Discussion of Counterfeit Banknote Detection

As mentioned above, unlike in the banknote recognition classification process, in which various classes have to be identified concurrently, counterfeit detection needs to classify only two classes, genuine banknotes and counterfeit banknotes, and the classifier may be accordingly considered less complicated.

However, the following two issues should be dealt with and solved in counterfeit banknote detection studies:

- Given the highly sophisticated techniques used for producing counterfeit banknotes, distinguishing them from genuine banknotes poses a great challenge. For counterfeit banknote classification, it is absolutely necessary to perform precise analyses of the characteristics of all anti-counterfeiting features (security features deliberately included in banknotes to deter counterfeiting) contained in the genuine banknotes of the denominations concerned.
- Counterfeit banknote detection is a perpetual process; if a highly efficient counterfeit detection algorithm is developed, more refined counterfeit banknotes disabling that algorithm appear, which necessitates the development of another algorithm to detect them in a never-ending spear-and-shield fight. For this reason, it is practically impossible to design a 100% perfect long-lasting counterfeit detection algorithm with a genuine banknote false rejection rate of 0%. As an alternative approach, developing a highly efficient counterfeit detection algorithm that would increase the counterfeit banknote production costs to such an extent that it is not worth making counterfeit banknotes may put an end to this endless combat.

## 4. Serial Number Recognition

### 4.1. Overall Procedure of Serial Number Recognition

**Figure 17:**
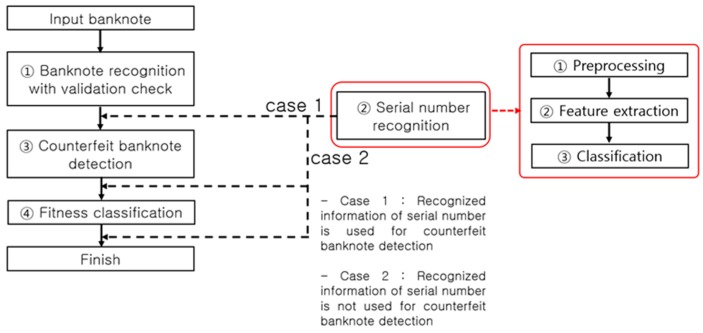
Serial number recognition process flow.

The serial number recognition step follows the banknote recognition step. It is carried out by performing image recognition of the ROI for the serial number specific to the denomination concerned using its position information based on the banknote recognition data. As shown in Figure 17, the serial number recognition process also goes through the subprocess steps of image preprocessing, feature extraction, and classification.

Like banknote recognition and counterfeit banknote detection, serial number recognition may have different degrees of accuracy depending on the currency type. Studies on serial number recognition can be divided into studies on the Chinese and Indian national currencies, as listed in Table 10.

In this table, “N/I” means the case that the detail information is not shown in the paper. “A” represents the case that the database is available for research purpose whereas “N/A” shows the case that the database is not available. In Table 10, the largest numbers of images are included in the database of [122]. Most databases are not available except for those in [126,127].

### 4.2. Image Preprocessing

**Figure 18:**
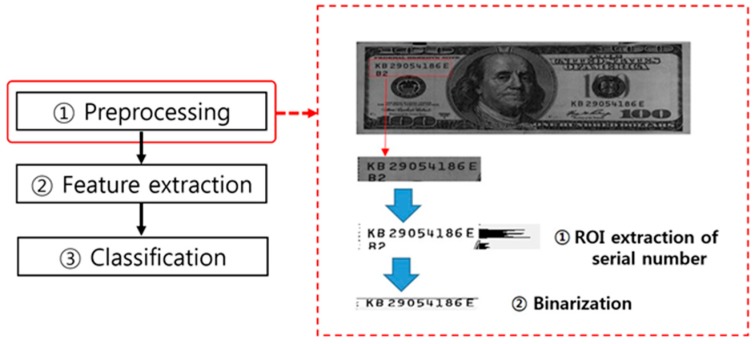
Example of image preprocessing in the serial number recognition process flow.

Following the banknote recognition process, the preprocessing step of serial number recognition generally undergoes noise-removal and binarization processes on the serial number ROI, as illustrated in Figure 18. Segmentation separating the serial number components from the background is performed as preprocessing for feature extraction to distinguish the individual letters/numbers of a serial number.

Table 11 lists the methods used in the preprocessing step of serial number recognition. If a banknote is in circulation for a long time, it may be difficult to distinguish its serial number from the background due to its surface being soiled by dirt and sebum from users’ hands. To address this, brightness normalization of the banknote image is performed using various methods such as mean filtering-based noise reduction [122,123], adjustment of brightness, contrast, and gamma [129], and gray-scale normalization [126]. Other measures include size normalization by bilinear interpolation [124] and binarization based on the area-ratio and block contrast [125]. These preprocessing measures render a banknote fit for separating the serial number from the background. Each letter/number of a serial number can then be distinguished from the background with prior information regarding the horizontal and vertical lengths of each letter/number in the serial number and a method to detect the outermost boundary positions of the horizontal and vertical directions of each letter/number [125].

### 4.3. Feature Extraction

**Figure 19:**
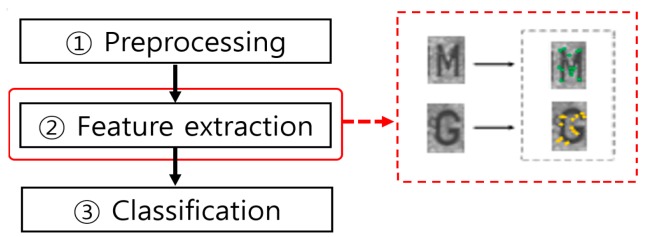
Example of key-point-based feature extraction in the serial number recognition process flow.

After preprocessing, serial number feature extraction is carried out on each ROI extracted as an individual letter/number, as illustrated in Figure 19, which presents an example of key-point-based feature extraction.

Serial number feature extraction is carried out using the methods presented in Table 12, namely extraction of features from nine local regions and four key-point features from each number/letter region [122] and extraction of gradient direction features [126]. Compared with the aforementioned feature extraction methods for banknote recognition and counterfeit banknote detection, there are only a limited number of studies on feature extraction for serial numbers. This may be attributed to the fact that serial number recognition can be classified, once the letter/number region of the serial number is detected, using a gray or binarized image, without going through the feature extraction step requiring a separate processing time because there are only letters/numbers and background in the serial number region.

### 4.4. Classification

**Figure 20:**
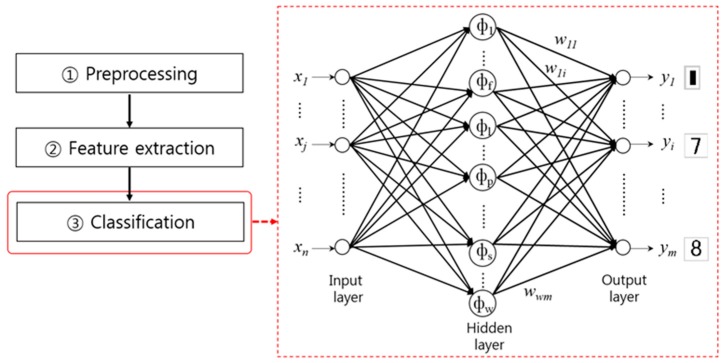
Example of NN-based classification in the serial number recognition process step.

Classification is carried out after determining the classes of the extracted the letters/numbers comprising the serial number based on their characteristics. Figure 20 illustrates an example of NN-based classification of a serial number.

As listed in Table 13, algorithms for classification used in the last step of the serial number recognition process step include the Euclidean distance-based matching method [122], SVMs [123], NNs [124,128], and a cascaded combination of multiple classifiers [126].

### 4.5. Analyses and Discussion of Serial Number Recognition

The following issues should be considered in banknote serial number recognition studies:

- While serial number recognition is methodologically similar to other in-document number recognition problems, it differs from them in that banknote surfaces get soiled over time due to dirt and sebum from users’ hands, making it increasingly difficult to distinguish the serial number from the background surface as a banknote ages. Moreover, banknotes are frequently exposed to risks of damage, such as creases and tears. This makes it necessary to design a strong system capable of serial number recognition on the images of various conditions of banknotes, including those heavily soiled with hand sebum and dirt or tattered with creases and tears.
- In general, a banknote serial number contains the year of printing and information on the issuing bank. Such information can be effectively used for tracing stolen money and detecting counterfeit banknotes once a denomination-wise banknote management system is established.

## 5. Fitness Classification

### 5.1. Overall Procedure of Fitness Classification

**Figure 21:**
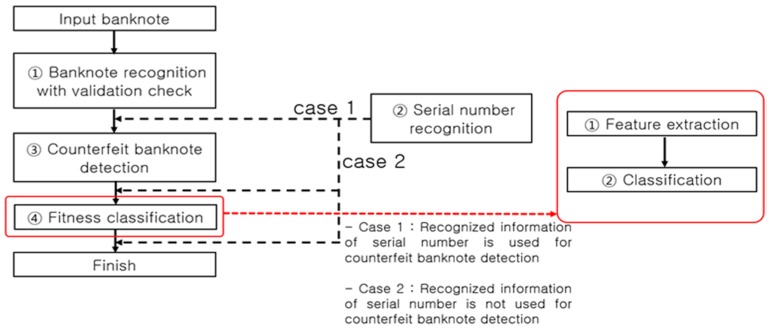
Process flow of fitness classification.

Fitness classification of a banknote generally refers to a technique to assess its soiling level using visible light and NIR image information. Unlike the image of a clean banknote, a banknote soiled by users’ hand dirt or sebum as the result of long use shows decreased brightness on a visible light or NIR image. A variety of factors influence the physical state of a banknote, such as the aforementioned soiling through dirt and sebum of users’ hands, creases, tears, and scribbling. Most of the previous studies on fitness classification use the soiling level as the criterion for judging a banknote fit or unfit for further circulation. As shown in Figure 21, fitness classification is carried out in the order of feature extraction and classification.

The standards and accuracy of fitness classification vary according to the currency. Table 14 shows that studies have been conducted on fitness classification of EUR, INR, CNY, USD, and KRW. In this table, “A” represents the case that the database is available for research purpose whereas “N/A” shows the case that the database is not available. In Table 14, the largest numbers of images are included in the database of [137]. Most databases are not available except for that in [138].

### 5.2. Feature Extraction

**Figure 22:**
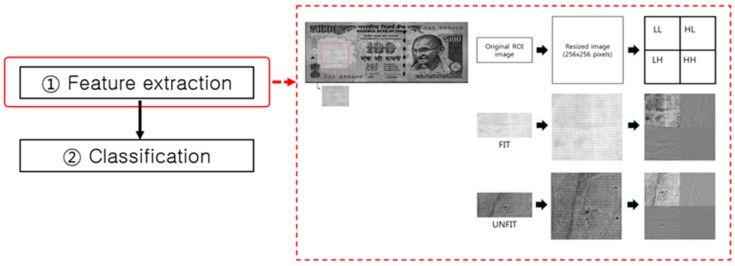
Feature extraction in the fitness classification process flow.

There are various feature extraction methods for fitness classification based on the aforementioned basic assumption that as the soiling level of a banknote increases, its image tends to be less bright, occasionally showing a higher dispersion rate. As shown in the example in Figure 22, a DWT can be used to extract such features for fitness classification.

Table 15 lists various methods that have been used for feature extraction for fitness classification, such as gray pixel value [133], color pixel value [133,135,136], pixel values of visible light and NIR images [100,134,138], gray level histogram [139], mean and standard deviation from the ROI by DWT [137], and acoustic features [141,142]. All methods, except for that using acoustic features, use visible light or NIR reflection or transmission images. The study on acoustic features adopted an approach to fitness verification using changes in acoustic features of a banknote, drawing on the fact that a banknote soiled by dirt and sebum from human hands becomes thicker. One of the listed studies presented a method using NIR transmission images instead of reflection images [138].

### 5.3. Fitness Classification

**Figure 23:**
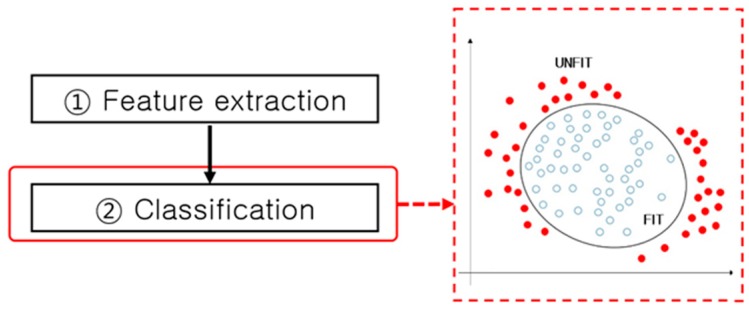
Classification in the fitness classification process flow.

The final fitness classification can be performed in the classification step using various types of classifier similar to that of the banknote recognition and serial number recognition processes. A method using an SVM-like nonlinear classifier, as shown in Figure 23, was also studied.

As listed in Table 16, methods used for the classification step of the fitness classification process include the Adaboost classifier [133,136], NNs [100,139], SVMs [137,140], and fuzzy systems [138]. As in the counterfeit detection process, banknotes are classified into two classes: fit and unfit.

### 5.4. Analyses and Discussion of Fitness Classification

Fitness classification of banknotes has been a neglected research area in comparison with banknote recognition and counterfeit banknote classification. However, with increasing penetration of automated self-service machines, such as ATMs, mechanical breakdown caused by unfit banknotes has become a serious maintenance and repair issue. Banknotes with high soiling levels also pose problems to banknote counters because they trigger false recognition and false rejection problems in banknote recognition, counterfeit banknote detection, and serial number recognition. Banks ensure circulation of only fit banknotes by withdrawing unfit banknotes from circulation by means of continuous fitness classification. In this context, fitness classification of banknotes has recently been attracting more attention. The following two issues should be considered in banknote fitness classification studies:

- Most methods for fitness classification classify banknotes into two classes: fit and unfit banknotes. However, such a binary classification has the inherent problem of requiring subjective judgment without any clear-cut quantifiable criteria. Therefore, experts are usually involved to perform visual assessment of the soiling level of banknotes, or densitometers are used to distinguish fit and unfit banknotes depending on the measured values.
- Besides the binary classification of fit and unfit banknotes, it is also important to ensure reproducibility of the assigned fitness level when the same banknote is put into a machine repeatedly.

## 6. Conclusions

Automated machines such as ATMs and banknote counters are indispensable for large-scale banknote circulation and safe transactions. Recent years have also seen extensive research on banknote recognition for visually impaired persons [18,23,42,52,143]. While many studies have been conducted on banknote recognition methods [143,144,145,146,147,148,149,150,151,152,153] or counterfeit banknote detection methods [97,120], this study provides an overview of the overall banknote recognition systems and describes in detail each of the process steps of banknote recognition, counterfeit banknote detection, serial number recognition, and fitness classification, listing the related studies and describing the methods presented by them. These methods are used to recognize banknote information (denomination, counterfeit detection, serial number, and fitness classification) using images or sensor data and can be applied to automated banknote dispensers and similar self-service terminals. As mentioned above, algorithms related to banknote recognition can show different performance characteristics for different currencies and numbers of target classes. The more similar the patterns among the denominations of a national currency and the higher the number of classes for classification, the lower the performance of the applied algorithm, and a stronger algorithm is required. However, an algorithm with a large number of computational operations cannot be efficiently used for devices requiring rapid processing in an embedded system environment without the aid of a PC, such as a banknote counter. Therefore, developing a banknote recognition algorithm with high recognition accuracy and processing speed is a great challenge for researchers. Besides banknote recognition, coin classification has recently been studied [154,155] using image-based size information and features robust to rotation.

The process flow of counterfeit banknote detection is similar to that of banknote recognition, including strong feature extraction and classification steps, but it requires a sensor responding to specific wavelengths in order to extract anti-counterfeiting features. While high-resolution image sensors and processors may be applied to extract microfine anti-counterfeiting features, such as anti-copier lines or microlettering, they are disadvantageous in terms of economic feasibility, and UV or NIR sensors are commonly used for analyzing anti-counterfeiting features. More recently, counterfeit banknote detection in a mobile environment has been studied [115,118], and a method using data communication with a server has also been presented [115]. Studies investigating counterfeit coin detection [89,90,156,157] mostly use the X-ray fluorescence-based component analysis instead of using visible light.

Serial number recognition is carried out after banknote recognition by determining the position information of the serial number specific to the denomination concerned as the ROI. It requires an image-processing technique capable of segmenting the serial number region from the background and a pattern-matching algorithm that can classify the class of each number/letter efficiently and rapidly. The serial number is a unique piece of information on each banknote, which lends itself well to counterfeit banknote detection. Additionally, if server-based data communication becomes possible for serial number management, it will greatly contribute to counterfeit banknote detection.

Fitness classification is a technique used for sorting banknotes in terms of soiling level and is necessary for ensuring circulation of clean banknotes. Fitness classification is also important from the perspective of counterfeit banknote detection because soiled banknotes tend to broaden the image range applicable to genuine banknotes, thus undermining the performance of banknote recognition and counterfeit perception algorithms. Fitness classification is also carried out in two process steps of feature extraction and classification. Its classification criteria are different from those of banknote recognition, counterfeit banknote detection, and serial number detection in that the results largely depend on the assessor’s own judgment. To address such an ambiguous boundary, a method employing a fuzzy system has been studied [138]. There is a need for a system that can freely control and define a fitness boundary.

As such, if the overall process flow of banknote recognition, counterfeit banknote detection, serial number recognition, and fitness classification could be carried out in a refined integral system, such a system would greatly contribute to safe banknote transactions and thus prove beneficial to society.

## Figures and Tables

**Table 1:** Studies on banknote recognition by national currency (Ref.: Reference(s), N/I: No Information, A: Available, N/A: Not Available).

Recognition Mode	National Currency	References	Databases			Availability of Database
			Ref.	#Images	#Denomination Kind	
Single Currency Recognition	United States (USD)	[8,9,15,16,17,18,19,20,21,22,23,24,25]	[8]	61,240	16	N/A
			[9]	99,236	17	N/A
			[15,22]	3570	6	N/A
			[16]	15,000	6	N/A
			[19]	65,700	12	N/A
	China (CNY)	[11,12,13,16,26,27,28,29,30,31]	[11]	297,200	3	N/A
			[13]	16,000	5	N/A
			[26]	3360	4	N/A
			[28]	20,000	5	N/A
			[30]	1600	4	N/A
	Euro (EUR)	[16,32,33,34,35]	[16]	15,000	7	N/A
			[32]	140	7	N/A
			[35]	82	N/I	N/A
	India (INR)	[36,37,38,39,40,41,42,43]	[36]	350	7	N/A
			[38]	39	3	N/A
			[41]	504	6	N/A
	South Korea (KRW)	[44]		10,800	3	N/A
	Iran (IRR)	[45,46,47,48]	[45]	4000	8	N/A
			[47]	128	8	N/A
			[48]	240	6	N/A
	Mexico (MXN)	[49,50]		1600	5	N/A
	Australia (AUD)	[51,52]	[51]	1320	6	N/A
	South African (ZAR)	[9]		760	10	N/A
	New Zealand (NZD)	[53]		367	5	N/A
	Sri Lanka (LKR)	[54]		280	4	N/A
	Pakistan (PKR)	[55]		120	6	N/A
	Angola (AOA)	[9]		1366	9	N/A
	Italy (ITL)	[56,57,58]	[57]	80	8	N/A
			[58]	30	8	N/A
	Saudi Arabia (SAR)	[37,59,60]	[37]	4	2	N/A
			[59]	300	3	N/A
			[60]	110	1	N/A
	Jordan (JOD)	[14]		500	10	A
	Ethiopia (ETB)	[61]		240	5	N/A
	Bangladesh (BDT)	[62,63]	[62]	1700	8	N/A
			[63]	N/I	7	N/A
	Myanmar (MMK)	[64]		89	5	N/A
	Malawi (MWK)	[9]		2464	6	N/A
Multi-Currency Simultaneous Recognition	USD, EUR, KRW, CNY, Russia (RUB)	[10]		100,797 from 5 national currencies	55 from 5 national currencies	N/A
	23 countries (CNY, EUR, INR, USD, etc.)	[65]		150 from 23 national currencies	101 from 23 national currencies	N/A
	Turkey (TRY), Cyprus (CYP)	[66]		180 (TRY), 144 (CYP)	5 (TRY), 4 (CYP)	N/A
	USD, EUR	[67]		N/I	4 (USD), 7 (EUR)	N/A
	USD, Japan (JPY)	[68]		132 (USD), 50 (JPY)	6 (USD), 3 (JPY)	N/A
	JPY, ITL, Spain (ESP), France (FRF)	[69]		165 (JPY), 440 (ITL), 385 (ESP), 275 (FRF)	3 (JPY), 8 (ITL), 7 (ESP), 5 (FRF)	N/A
	USD, EUR, BDT, INR	[70]		300 (USD), 300 (EUR), 500 (BDT), 300 (INR)	3 (USD), 3 (EUR), 5 (BDT), 3 (INR)	N/A

**Table 2:** Methods for preprocessing in the banknote recognition process flow.

Task	Method	References
Banknote region segmentation	Corner detection	[8,9]
	Least square method and fuzzy system	[12]
	Component labeling based on the Y component of YIQ space	[68]
Noise removal and gray level reduction	Weiner filtering	[10,49,55,65,71]
	Median filtering	[42,64]
	Gray level reduction	[54,65,71,72]
Brightness normalization and contrast enhancement	Histogram equalization	[42,45]
Image resolution reduction	Nearest neighbor interpolation	[10,67]
Image channel reduction	Conversion of color to gray	[23,39,46,55,63,72]

**Table 3:** Methods for feature extraction in the banknote recognition process flow.

Method	References
Features of banknote size or length	[45,56,60,65,68,72]
Color information (RGB, HSV, or HSI)	[37,40,45,49,50,61,68]
Edge information (Canny, Prewitt, or Sobel operator)	[40,44,54,60]
Histogram information (correlation, central moments, kurtosis, mean, standard deviation, skewness, etc.)	[39,43,53,59,64,65]
Local binary patterns (LBP)	[41,49]
Gray-level co-occurrence matrix (GLCM)	[39,53,64]
Principle component analysis (PCA)	[8,9,15,20,21,22,23,26,46]
Linear discriminant analysis (LDA)	[43,46,70]
Genetic algorithm (GA)	[24,30,69,73]
Similarity map or difference map	[9,10,19]
Discrete wavelet transform (DWT)	[11,16,44,47,48]
Scale-invariant feature transform (SIFT) or speeded up robust features (SURF)	[14,17,18,25,35,36,61,67,74]
Compressed sensing	[27]
Features by optical character recognition (OCR)	[38]
Features from selected ROI	[8,9,13,17,20,23,30,32,36,38,39,43,48,59,68]

**Table 4:** Studies on classification and verification in the banknote recognition process flow.

Methods		References
Classification	Euclidean distance-based classifier	[36,37,41,42,48,51]
	Mahalanobis distance-based classifier	[23]
	NN (LVQ network, ENN and PNN, etc.)	[15,16,20,21,22,24,26,29,30,31,32,33,34,38,45,46,47,49,53,54,56,57,58,62,63,66,69,72,73]
	SVM	[8,11,39,43,67,71]
	HMM	[13,65,71]
	K-means algorithm	[8,9]
	K-NN method	[55,64]
	Preclassification (based on banknote side, direction, size, or a Gaussian mixture model (GMM))	[8,10,32,75]
Verification	Verification (based on the validity of matching distance or banknote size)	[9,54]

**Table 5:** Feature and advantage analyses of existing banknote recognition methods (DSP processing, high-capacity DB, multi-currency simultaneous recognition).

References	Features and Advantages
[8]	Banknote counter DSP processing (processing time: 15.6 ms), preclassification of the banknote input side (SVM), number of experimental data points (61,240 notes), accuracy (USD: 99.886%)
[73]	Banknote counter DSP processing (banknote counting machine by Glory Corp.), GA-based selection of optimal mask and use of a NN, number of experimental data points (100,000 notes), accuracy (USD and JPY: ≥97%)
[9]	Banknote counter DSP processing, feature region selection using a similarity map, number of experimental data points (99,236 USD notes), accuracy (USD: 99.998%)
[10]	Simultaneous recognition of 5 national currencies (USD, EUR, KRW, CNY, RUB), ROI selection after using a similarity map, number of experimental data points (84,800 of 5 kinds of banknote), accuracy (100%)
[16]	Quaternion WT-based data extraction of the magnitude, horizontal, vertical, and diagonal data of banknote images and coefficient feature extraction using the generalized Gaussian density function, number of experimental data points (15,000 USD, CNY, EUR notes each), accuracy (≥99% on average)
[65]	Simultaneous recognition of 23 national currencies including USD, EUR, INR, and CNY, banknote texture feature modeling using size data and a HMM, number of experimental data points (150 per denomination), recognition rate (98%)
[67]	ATM DSP processing (processing time: 54 ms), simultaneous recognition of USD and EUR using the dense SIFT feature extraction method, accuracy (≥99.8%)
[19]	Real-time embedded system processing (processing time: 16 m), valid feature region selection using the difference map, generalized learning vector quantization (GLVQ) classification, number of experimental data points (65700 USD notes), accuracy (99%)
[69]	GA-based selection of optimal mask, NN-based DSP simultaneous recognition of four national currencies (JPY, ITL, ESP, FRF) using a banknote counter, number of experimental data points (20,000 notes), accuracy (97%)
[74]	Multi-currency simultaneous recognition (INR, CNY, EUR, etc.) using a mobile camera and server communication system with a feature enabling overlapping multi-currency simultaneous recognition, recognition rate (95%)

**Table 6:** Methods for identifying anti-counterfeiting features.

Feature	Method	References
Brightness information	Y histogram of YIQ color space or luminance histogram	[83,84]
Fluorescence characteristics	UV pattern	[85,86,87]
	X-Ray fluorescence	[88,89,90,91]
	Intrinsic fluorescence lifetime	[92]
Fidelity of serial number and printing	Binarization, edge detection, and radial based function (RBF) NNs	[93]
	Printing accuracy by tie point detection	[94]
Security thread	Electromagnetic detection based on the pulsed eddy current technique	[95]
Infrared (IR) features	The middle IR spectrum of several areas in the banknotes	[96]
	Near IR features	[75,82,97,98,99,100]
	Commercial system using multiple sensors including IR ray sensor	[101]

**Table 7:** Studies on counterfeit banknote detection by currency (Ref.: References, N/I: Not Informed, N/A: Not Available).

National Currency	References	Databases			Availability of Database
		Ref.	#Images	#Denomination Kind	
India (INR)	[84,87,99,102,103,104,105,106,107,108,109,110,111,112,113,114,115]	[87]	1000	2	N/A
		[113]	288	3	N/A
Euro (EUR)	[88,94,96,98]	[96]	18	2	N/A
		[98]	2750	7	N/A
United States (USD)	[88,91,92]	[91]	120	2	N/A
		[92]	10	5	N/A
Kuwait (KWD)	[116]		4	2	N/A
Nepal (NPR)	[117]		240	1	N/A
Switzerland (CHF)	[118]		82	2	N/A
Taiwan (TWD)	[83,119]	[83]	99	N/I	N/A
		[119]	200	N/I	N/A
South Korea (KRW)	[85,86]	[85]	360	3	N/A
		[86]	N/I	9	N/A
United Kingdom (GBP)	[95]		3	2	N/A
China (CNY)	[86]		N/I	1	N/A
Malaysia (MYR)	[86]		N/I	1	N/A

**Table 8:** Methods for feature extraction in the counterfeit banknote detection process flow.

Method	References
Features from intaglio printing, ink properties, artwork, fluorescence, or year of printing	[87,106]
Bit-plane slicing and Canny edge detection	[116]
Watermark segmentation	[105,106,108,110,113,114]
Luminance histograms and texture features from GLCM	[84]
DWT	[102]
Security thread information	[87,103,104,105,110,113,114]
Optically variable ink information	[106,110,113]
SIFT algorithm	[112]
Mean, standard deviation, skewness, entropy, and correlation in an ROI	[117]
Identification mark or number panels	[103,104,105,106]
Micro lettering or latent image	[104,106]

**Table 9:** Methods for classification in the counterfeit banknote detection process flow.

Method	References
Template matching or keypoint matching	[106,117]
Artificial NN	[121]
SVM	[84,87,119]
Multiple kernel SVM	[111]

**Table 10:** Resources on serial number recognition by currency (Ref.: References, N/I: No Information, A: Available, N/A: Not Available).

National Currency	References	Databases			Availability of Database
		Ref.	#Images	#Denomination Kind	
China (CNY)	[93,122,123,124,125,126,127,128]	[122]	40,000	2	N/A
		[125]	5000	N/I	N/A
		[126,127]	24,262	2	A
India (INR)	[129,130,131,132]	[129,130]	25	5	N/A

**Table 11:** Methods for preprocessing in the serial number recognition process flow.

Methods	References
Mean filtering for noise reduction	[122,123]
Adjustment of brightness, contrast, and gamma	[129]
Size normalization by bilinear interpolation	[124]
Binarization based on the area-ratio and block contrast	[125]
Gray-scale normalization	[126]

**Table 12:** Methods for feature extraction in the serial number recognition process step.

Method	References
Features from nine local regions and four key-point features	[122]
Gradient direction feature	[126]

**Table 13:** Methods for classification in the serial number recognition process flow.

Method	References
Euclidean distance-based matching	[122]
SVM	[123]
NN	[124,128]
Cascaded combination of multiple classifiers	[126]

**Table 14:** Studies on fitness classification by national currency (Ref.: References, A: Available, N/A: Not Available).

National Currency	References	Databases			Availability of Database
		Ref.	#Images	#Denomination Kind	
Euro (EUR)	[100,133,134,135,136]	[100]	800	4	N/A
		[133,136]	9029	4	N/A
India (INR)	[137,138]	[137]	19,300	5	N/A
		[138]	2300	6	A
China (CNY)	[139,140]	[140]	4400	1	N/A
United States (USD)	[138]		3856	7	A
South Korea (KRW)	[138]		3956	4	A

**Table 15:** Methods for feature extraction in the fitness classification process flow.

Method	References
Gray pixel value	[133]
Color pixel value	[133,135,136]
Pixel values of visible light and NIR images	[100,134,138]
Gray level histogram	[139]
Mean and standard deviation from ROI by DWT	[137]
Acoustic features of banknotes	[141,142]

**Table 16:** Methods for classification in the fitness classification process flow.

Method	References
Adaptive boosting (Adaboost) classifier	[133,136]
NN (RBF network or sine basis function)	[100,139]
SVM	[137,140]
Fuzzy system	[138]
